# Dietary phytogenic inclusion level affects production performance and expression of ovarian cytoprotective genes in laying hens

**DOI:** 10.1016/j.psj.2023.102508

**Published:** 2023-01-16

**Authors:** Ioannis P. Brouklogiannis, Evangelos C. Anagnostopoulos, Eirini Griela, Vasileios V. Paraskeuas, Konstantinos C. Mountzouris

**Affiliations:** Laboratory of Nutritional Physiology and Feeding, Department of Animal Science, School of Animal Biosciences, Agricultural University of Athens, 11855 Athens, Greece

**Keywords:** laying hen, phytogenic, ovary, performance, cytoprotective response

## Abstract

A 12-wk study was conducted to investigate the effects of a phytogenic premix (**PP**) inclusion level on production performance, and the expression of genes relevant for detoxification (aryl hydrocarbon receptor; **AhR**) and antioxidant capacity (Nuclear factor erythroid 2-related factor 2; **Nrf2**) in the ovaries of laying hens. The PP consisted of bioactive substances derived from ginger, lemon balm, oregano, and thyme substances (Anco FIT-Poultry). Depending on PP inclusion level (i.e., 0, 500, 750, 1,000, and 1,500 mg/kg diet) in the basal diet, 385 laying hens Hy-Line Brown, 20-wk-old were assigned into 5 treatments: CON, P500, P750, P1000, and P1500, with 7 replicates of 11 hens with ad libitum access to feed and water. Performance parameters were closely monitored on a weekly basis and analyzed in the following 3 experimental periods: 1 to 4 wk, 5 to 8 wk, and 9 to 12 wk of treatment administration (i.e., 21–24, 25–28, and 29–32 wk of layers age, respectively). At the end of the 8th and 12th wk of the experiment (i.e., 28 and 32 wk of layers age), a layer from each replicate was selected, euthanized, the ovaries sampled and stored deep frozen until gene expression analysis. Data were analyzed by ANOVA and means compared using Tukey's honest significant difference test. Polynomial contrasts tested the linear and quadratic effect of PP inclusion levels. Results revealed that PP inclusion, improved (*P* < 0.05) laying rate and egg mass, compared to CON. Increasing PP inclusion level enhanced laying rate and egg mass, linearly and quadratically and peaked at P1000 (*P* < 0.05). In the ovaries, the AhR pathway genes assessed were down-regulated (*P* < 0.05) mainly at P1000 and P750 treatments. In addition, PP related cytoprotective potential was demonstrated via beneficial changes seen for the majority of the Nrf2-pathway genes assessed with the P1000 displaying most significant differences from CON. Conclusively, new data highlighted beneficial cytoprotective effects of PP inclusion on layer ovaries and documented further layer performance, with the inclusion level of 1000 mg PP/kg diet being the most prominent.

## INTRODUCTION

The ovary is a critical organ of the reproductive system of laying hens that is strongly associated with reproductive performance and its dysfunction can lead to reduced ovulation, egg production and commercial value of the layers. Highly productive laying hens are more vulnerable to oxidative stress and ovarian aging due to rapid daily ovulations (Liu et al., 2018a; Dai et al., 2021; Zhang et al., 2022). Intensive poultry production is associated with a variety of nutritional, environmental, and management factors that can lead to oxidative stress. The latter is an imbalance of free radicals such as reactive oxygen species (**ROS**), reactive nitrogen species and antioxidant defense in cells and tissues. If not controlled, oxidative stress may result in lipid peroxidation, protein oxidation, DNA damage, apoptosis and inflammation, which negatively impact birds’ productive and reproductive performance, health and product quality (Sahin et al., 2013; Pisoschi and Pop, 2015; Lee et al., 2019; Lauridsen, 2019; Mountzouris et al., 2022).

Nutritional application of bioactive feed additives such as phytogenics (i.e., aromatic plants, herbs, spices, essential oils and their bioactive components), present a plausible strategy to control and manage the production of ROS. Phytogenic feed additives have been used to enhance the productive performance of poultry and their cellular defense against ROS due to their antioxidant and anti-inflammatory properties (He et al., 2017; Lee et al., 2019; Abdel-Wareth and Lohakare, 2020; Zhang et al., 2022). More specifically, dietary phytogenics may activate two signaling pathways, namely the aryl hydrocarbon receptor (**AhR**) and the nuclear factor erythroid 2-related factor 2 (**Nrf2**), which are essential for cellular protection. In particular, the AhR pathway is responsible for detoxification and the Nrf2 is responsible for the adaptive antioxidant defense and overall cytoprotection (Köhle and Bock, 2006; Seymour et al., 2013; Mountzouris et al., 2022)

Recent studies indicate that dietary phytogenics could function as AhR/Nrf2 modulators. For example, phytogenic inclusion induced protective responses against oxidative stress through modulation of target AhR pathway genes in the liver of broilers and piglets (Muhammad et al., 2017; Ates and Ortatatli, 2021; Xun et al., 2021). In laying hens the activation of the Nrf2 pathway and it's antioxidant genes by phytogenics, attenuated oxidative stress before laying, at the peak laying phase (Xing et al., 2020; Zhang et al., 2022) and also during the ovarian aging process (Liu et al., 2018a; b). Furthermore, at the protein level phytogenics increased the concentration of Nrf2 in aged breeder hens improving the ovarian function, contributing to the maintenance of reproductive health (Dai et al., 2021).

Besides to the limited information regarding the Nrf2 pathway response in the ovary of layers fed with phytogenics, currently there are no studies reporting respective data on the AhR pathway. Moreover, there is still limiting scientific information on the physiological AhR/Nrf2 pathway response in the ovaries and its relevance for the layer production responses. The aim of this study was to assess critical AhR/Nrf2 pathway gene components in the ovaries of layers fed diets with increasing phytogenic inclusion level. In this respect, layer performance and the expression of 18 critical genes relevant for detoxification (AhR pathway) and antioxidant capacity (Nrf2 pathway) in the ovaries of young laying hens were assessed during the first 12-wk of peaking phase.

## MATERIALS AND METHODS

### Animals, Housing and Experimental Treatments

Practices regarding the care and use of animals for research purposes were in accordance with the institutional and national guidelines and approved (Protocol number: 33/24072020) by the Bioethics Committee of the Agricultural University of Athens (**AUA**), Greece. Following an initial 7-wk rearing period at the AUA laying hen facility, 385 commercial Hy-Line Brown laying hens (20-wk-old), with uniform body weight and similar performance were allocated into 5 treatments with 7 replicates of 11 hens each, for a 12-wk experimental period. Depending on the level of a phytogenic premix (**PP**) inclusion in a corn-soybean meal basal diet, dietary treatments included: control (**CON**) basal diet without PP, and basal diets containing PP at 500 (**P500**), 750 (**P750**), 1000 (**P1000**) and 1500 (**P1500**) mg/kg diet, respectively. The PP used in this trial (Anco FIT–Poultry, Anco Animal Nutrition Competence, GmbH, Sankt Poelten, Austria) was a proprietary mixture of phytogenic substances marketed as a “gut agility activator” with an active ingredient concentration of 70 g/kg. The PP consisted of bioactive substances derived from ginger, lemon balm, oregano and thyme on functional carriers 1m558 bentonite and clinoptilolite. All diets were formulated to meet or exceed the recommendations provided in Hy-line brown Management Guide (2018). The calculated chemical composition per kilogram of the basal diet was as follows: ME 2915.9 kcal (12.2 MJ), CP 165 g, calcium 41.0 g, and available phosphorus 4.8 g. Feed, in mash form, and water were provided ad libitum during the experiment. Hens were kept in 3-floor battery cages (192 cm length × 45 cm width × 48 cm height) under controlled environmental conditions, and a gradual increasing light photoperiod, in order to achieve 16 h of light (16L:8D) at approximately 30 weeks of age, following the Light Program for Light provided in Hy-line brown Management Guide (2018).

### Laying Performance

Eggs produced were collected and weighted daily. The number of the eggs and average egg weight were recorded. The laying rate was determined each week as the total number of eggs divided by 7 d. Feed intake was recorded on a weekly basis. Egg mass was calculated by multiplying average egg weight by laying rate. Feed conversion ratio was calculated as grams of feed intake per gram of egg mass. Data regarding laying rate, egg mass, feed intake, and feed conversion ratio were reported as average values on a 4 wk basis (i.e., wk1–4; wk 5–8, and wk 9–12). The mortality was recorded daily throughout the experimental period.

### Tissue Sampling for Subsequent Analyses

At the end of the 8th and 12th experimental week (i.e., 28 and 32 wks of layers age), 7 hens per treatment were randomly selected, anaesthetized (EC 1099/2009) and euthanized by severing the jugular vein. Subsequently, ovary samples were carefully excised aseptically, snap frozen in liquid nitrogen, and subsequently stored at −80°C for further analysis.

### RNA isolation and reverse transcription to cDNA

For the determination of target gene expression, total RNA from ovarian samples was isolated by using NucleoZOL Reagent (Macherey-Nagel GmbH & Co. KG, Düren, Germany), according to the manufacturer's protocol. Subsequently, RNA quality and quantity were assessed by spectrophotometry with the use of NanoDrop-1000 (Thermo Fisher Scientific, Waltham, United Kingdom) followed by DNase treatment. In particular, 10 μg of RNA were diluted with 1 U of DNase I (M0303, New England Biolabs Inc, Ipswich, UK) and 10 μL of 10x DNAse buffer to a final volume of 100 µL with the addition of DEPC water for 20 min at 37°C. Prior to DNAse inactivation at 75°C for 10 min, 1 μL of 0.5 mol/L EDTA was added to protect RNA from being degraded during enzyme inactivation. RNA integrity was assessed by agarose gel electrophoresis. For cDNA preparation, 500 ng of total RNA from each sample were reverse transcribed to cDNA by PrimeScript RT Reagent Kit (Perfect Real Time, Takara Bio Inc., Shiga-Ken, Japan) following the manufacturer's guidelines. All cDNAs were afterwards stored at −20°C.

### Quantitative Real-Time PCR Analysis

The following *Gallus gallus* genes were examined: aryl hydrocarbon receptor 1 (***Ahr1***), aryl hydrocarbon receptor nuclear translocator (***ARNT***), cytochrome P450 1A1 (***CYP1A1***), cytochrome P450 1A2 (***CYP1A2***), cytochrome P450 1B1 (***CYP1B1***), glutathione S-transferase alpha 2 (***GSTA2***), NAD(P)H quinone dehydrogenase 1 (***NQO1***), nuclear factor erythroid-derived 2-like 2 (**Nrf2**), kelch like ECH associated protein 1 (***Keap1***), catalase (***CAT***), superoxide dismutase 1 (***SOD1***), glutathione peroxidase 2, 7 (***GPX2, GPX7***), glutathione-disulfide reductase (***GSR***), peroxiredoxin-1 (***PRDX1***), heme oxygenase 1 (***HMOX1***), heat shock protein 70 (***HSP70***), and heat shock protein 90 (***HSP90***). Suitable primers were designed using the GenBank sequences deposited on the National Center for Biotechnology Information and US National Library of Medicine (**NCBI**) shown in Table 1. Primers were checked using the PRIMER BLAST algorithm for *Gallus gallus* mRNA databases to ensure that there was a unique amplicon. Real-time quantitative PCR (**qPCR**) was accomplished in 96-well microplates with a SaCycler-96 Real-Time PCR System (Sacace Biotechnologies s.r.l., Como, Italy) and FastGene IC Green 2x qPCR universal mix (Nippon Genetics, Tokyo, Japan). Every reaction included 12.5 ng RNA equivalents along with 200 nmol/L of forward and reverse primers for each gene. The reactions were incubated at 95°C for 3 min, accompanied by 40 cycles of 95°C for 5 s, 59.5 to 62°C (depending on the target gene) for 20 s, 72°C for 33 s. This was tailed by a melt curve analysis to check the reaction specificity. Each sample was measured in duplicates. Relative expression ratios of target genes were calculated according to Pfaffl (2001) adapted for the multi-reference genes normalization procedure according to Hellemans et al. (2008) using *GAPDH* and *ACTB* as reference genes.

**Table 1:** Oligonucleotide primers used for gene expression of selected targets by quantitative real time PCR.

Gene	Primer sequence (5′-3′)^2^	Annealing temperature (°C)	PCR product size (bp)	GenBank (NCBI Reference Sequence)
GAPDH	F: ACTTTGGCATTGTGGAGGGT R: GGACGCTGGGATGATGTTCT	59.5	131	NM_204305.1
ACTB	F: CACAGATCATGTTTGAGACCTT R: CATCACAATACCAGTGGTACG	60	101	NM_205518.1
AhR pathway				
AhR1	F: TTTAGTGTGGCAGGTGGATT R: CCTTGTGCCAATGATGCTATTTG	60	200	NM_204118.2
ARNT	F: GAGACCAAGGCCCCAACTAC R: TCGGGTGCCTCTTTCTTTCC	62	140	NM_204200.1
CYP1A1	F: GTGATGGAGGTGACCATCGG R: ACATTCGTAGCTGAACGCCA	62	165	NM_205147.1
CYP1A2	F: CTGACCGTACACCACGCTT R: CTCGCCTGCACCATCACTTC	62	75	NM_205146.2
CYP1B1	F: CAGTGACTCCGCATCCCAAA R: CCATACGCTTACGGCAGGTT	62	132	XM_015283751.2
GSTA2	F: GCCTGACTTCAGTCCTTGGT R: CCACCGAATTGACTCCATCT	60	138	NM_001001776.1
NQO1	F: GAGCGAAGTTCAGCCCAGT R: ATGGCGTGGTTGAAAGAGGT	60.5	150	NM_001277619.1
Nrf2 pathway				
Nrf2	F: AGACGCTTTCTTCAGGGGTAG R: AAAAACTTCACGCCTTGCCC	60	285	NM_205117.1
Keap1	F: GGTTACGATGGGACGGATCA R: CACGTAGATCTTGCCCTGGT	62	135	XM_025145847.1
CAT	F: ACCAAGTACTGCAAGGCGAA R: TGAGGGTTCCTCTTCTGGCT	60	245	NM_001031215
SOD1	F: AGGGGGTCATCCACTTCC R: CCCATTTGTGTTGTCTCCAA	60	122	NM_205064.1
GPX2	F: GAGCCCAACTTCACCCTGTT R: CTTCAGGTAGGCGAAGACGG	62	75	NM_001277854.1
GPX7	F: GGCTCGGTGTCGTTAGTTGT R: GCCCAAACTGATTGCATGGG	60	139	NM_001163245.1
GSR	F: GTGGATCCCCACAACCATGT R: CAGACATCACCGATGGCGTA	62	80	XM_015276627.1
HMOX1	F: ACACCCGCTATTTGGGAGAC R: GAACTTGGTGGCGTTGGAGA	62	134	NM_205344.1
PRDX1	F: CTGCTGGAGTGCGGATTGT R: GCTGTGGCAGTAAAATCAGGG	61	105	NM_001271932.1
Heat shock proteins				
HSP70	F: ATGCTAATGGTATCCTGAACG R: TCCTCTGCTTTGTATTTCTCTG	60	145	NM_001006685.1
HSP90	F: CACGATCGCACTCTGACCAT R: CTGTCACCTTCTCCGCAACA	60	196	NM_001109785.1

ACTB, actin beta; AhR1, aryl hydrocarbon receptor 1; ARNT, aryl hydrocarbon receptor nuclear translocator; CAT, catalase; CYP1A1, cytochrome P450 1A1; CYP1A2, cytochrome P450 1A2; CYP1B1, cytochrome P450 1B1; GAPDH, glyceraldehyde 3-phosphate dehydrogenase; GSR, glutathione-disulfide reductase; GST++A2, glutathione S-transferase alpha 2; GPX2,7, glutathione peroxidase 2, 7; HMOX1, heme oxygenase 1; HSP70, heat shock 70 kDa protein; HSP90, heat shock protein 90 alpha family class A member 1; Keap1, kelch-like ECH-associated protein 1; NQO1, NAD(P)H quinone dehydrogenase 1; Nrf2, nuclear factor; erythroid 2-like 2; PRDX1, peroxiredoxin-1; SOD1, superoxide dismutase 1.

### Statistical Analysis

Experimental data on layer performance were based on a cage basis, whereas ovarian gene expressions were based on individual layers. All data were initially checked for normality and subsequently analyzed with the general linear model (**GLM**)–ANOVA procedure using the SPSS for Windows statistical package program, version 27 (SPSS Inc., Chicago, IL). Statistically significant effects were further analyzed, and means were compared using Tukey's honest significant difference (**HSD**) multiple comparison procedure. Statistical significance was determined at *P* ≤ 0.05. Linear (**lin**) and quadratic (**quad**) response patterns to dietary PP inclusion level were studied using polynomial contrasts.

## RESULTS

### Productive Performance

The effects of PP inclusion on productive performance responses are shown in Table 2. There were no (*P* > 0.05) differences between the experimental treatments for any of the performance parameters studied in the period 1 to 4 wk of the experiment (i.e., 21–24 wk of layers age). Dietary inclusion of PP significantly (*P* < 0.05) increased laying rate and egg mass in the periods 5 to 8 wk and 9 to 12 wk of the experiment (25–28 and 29–32 wk of layers age, respectively). In period 5 to 8 wk (25–28 wk of layers age), laying rate increased in a linear (*P*_lin_ = 0.029) and a quadratic (*P*_quad_ < 0.001) fashion with increasing PP inclusion level with P500, P750, and P1000 being higher than control. Also, egg mass displayed a quadratic (*P*_quad_ = 0.033) pattern of increase with increasing PP inclusion level with P1000 being higher than CON. In the last period 9 to 12 wk of the experiment (29–32 wk of layers age), all PP experimental treatments had highly (*P* < 0.001) higher laying rate and egg mass compared to CON. Likewise, increasing PP inclusion level resulted in linear (*P*_lin_ < 0.001) and quadratic (*P*_quad_ < 0.001) patterns of increase for laying rate and egg mass. More specifically, laying rate and egg mass in treatment P1000 were higher compared primarily to control and secondarily to P1500. There was no mortality throughout the experiment.

**Table 2:** Effect of dietary PP inclusion level on the laying performance.

Component	Treatments^1^						Statistics^2^		
Laying rate %	CON	P500	P750	P1000	P1500	SEM^3^	*P*_anova_	*P*_linear_	*P*_quadratic_
1–4 wk	94.8	94.5	95.7	95.9	93.7	1.38	0.535	0.796	0.220
5–8 wk	96.0^B^	98.1^A^	98.4^A^	98.8^A^	97.2^AB^	0.63	0.001	0.029	<0.001
9–12 wk	95.6^C^	98.0^B^	99.0^AB^	100.0^A^	97.5^B^	0.54	<0.001	<0.001	<0.001
Egg mass g/hen/d									
1–4 wk	52.4	52.7	52.4	53.1	51.1	1.04	0.389	0.362	0.196
5–8 wk	57.0^b^	59.1^ab^	58.2^ab^	59.3^a^	58.1^ab^	0.78	0.040	0.158	0.033
9–12 wk	58.6^B^	60.8^AB^	60.6^B^	62.2^A^	60.7^B^	0.50	<0.001	<0.001	<0.001
Feed intake g/hen/d									
1–4 wk	102.8	105.8	103.8	105.3	103.8	1.61	0.351	0.685	0.199
5–8 wk	109.7	111.9	111.8	112.5	111.8	1.54	0.427	0.163	0.225
9–12 wk	111.4	113.9	113.2	113.0	114.0	1.56	0.468	0.221	0.564
Feed conversion ratio									
1–4 wk	1.96	2.01	1.98	1.98	2.03	0.038	0.475	0.219	0.755
5–8 wk	1.93	1.89	1.92	1.90	1.92	0.028	0.622	0.964	0.346
9–12 wk	1.90	1.87	1.87	1.82	1.88	0.028	0.059	0.140	0.067

1 PP Inclusion (CON = 0 mg/kg, P500 = 500 mg/kg, P750 = 750 mg/kg, P1000 = 1000 mg/kg, and P1500 = 1500 mg/kg of diet). Data represent treatment means from n = 7 replicates per treatment.

2 Means with different superscripts (a, b, c or A, B, C) within the same row differ significantly (*P* < 0.05 or 0.01).

3 Standard error of the means. ^4^Reference to 1 to 4 wk, 5 to 8 wk, and 9 to 12 wk of experimental period correspond to 21 to 24, 25 to 28, and 29 to 32 wk of layers age, respectively.

### mRNA transcript levels of AhR pathway genes in Ovaries

#### Week 8

In the ovaries, the relative expression levels of *AhR* pathway (*AhR1, ARNT, CYP1A1, CYP1A2, CYP1B1, NQO1,* and *GSTA2*) related genes at wk 8 and wk 12 (28 and 32 wk of layers age) are presented in Tables 3 and 4, respectively. The inclusion of PP significantly changed (*P* < 0.05) the relative expression of *ARNT* (*P* = 0.003), *CYP1A1* (*P* = 0.050), *CYP1B1* (*P* < 0.001) and *GSTA2* (*P* = 0.006) between the experimental treatments at week 8 of the experiment (28th wk of layers age). Polynomial contrast analysis showed that the expression of *ARNT* (*P*_quad_ < 0.001), *CYP1A1* (*P*_quad_ = 0.006), *CYP1A2* (*P*_quad_ = 0.015), and *CYP1B1* (*P*_quad_ < 0.001) displayed quadratic patterns of decrease with increasing PP inclusion level. The expression of *GSTA2* displayed quadratic (*P*_quad_ = 0.004) and linear (*P*_lin_ = 0.025) patterns of increase. Increasing PP inclusion level resulted in reduced expression, in a linear pattern for *CYP1A1* (*P*_lin_ = 0.050) and *CYP1B1* (*P*_lin_ < 0.001). Compared to control treatment, the relative expression of *ARNT* was lowest for P500 and of *CYP1A1* was lowest for PP treatments 750 and 1000) at the same time. As for *CYP1B1 and GSTA2* expression levels*,* P750 treatment displayed the lowest and P1000 the highest compared to CON, respectively.

**Table 3:** Relative gene expression of AhR pathway related genes in layer ovaries at 8th week (28 wk old layers) of the experiment.

AhR pathway	Treatments^1^						Statistics^2^		
Genes (28 wk old layers)	CON	P500	P750	P1000	P1500	SEM^3^	*P*_anova_	*P*_linear_	*P*_quadratic_
AhR1	1.20	0.91	1.04	0.92	0.92	0.155	0.266	0.109	0.434
ARNT	2.62^A^	0.86^C^	0.93^BC^	1.65^ABC^	2.40^AB^	0.514	0.003	0.755	<0.001
CYP1A1	1.63^a^	0.92^ab^	0.85^b^	0.85^b^	1.09^ab^	0.251	0.020	0.050	0.006
CYP1A2	1.44	0.71	0.81	0.57	1.85	0.489	0.140	0.983	0.015
CYP1B1	2.15^A^	0.83^B^	0.75^B^	0.82^B^	0.84^B^	0.242	<0.001	<0.001	<0.001
GSTA2	0.65^B^	1.17^AB^	1.51^AB^	1.92^A^	1.11^AB^	0.316	0.006	0.025	0.004
NQO1	1.09	1.08	1.01	1.02	1.07	0.092	0.863	0.603	0.426

1 PP Inclusion (CON = 0 mg/kg, P500 = 500 mg/kg, P750 = 750 mg/kg, P1000 = 1000 mg/kg, and P1500 = 1500 mg/kg of diet). Data represent treatment means from n = 7 replicates per treatment.

2 Means with different superscripts (a, b, c or A, B, C) within the same row differ significantly (*P* < 0.05 or 0.01).

3 Standard error of the means. AhR1, aryl hydrocarbon receptor 1; ARNT, aryl hydrocarbon receptor nuclear translocator; CYP1A1, cytochrome P450 1A1; CYP1A2, cytochrome P450 1A2; CYP1B1, cytochrome P450 1B1; GSTA2, glutathione S-transferase alpha 2; NQO1, NAD(P)H quinone dehydrogenase 1.

**Table 4:** Relative gene expression of AhR pathway related genes in layer ovaries at 12th week (32 wk old layers) of the experiment.

AhR pathway	Treatments^1^						Statistics^2^		
Genes (32 wk old layers)	CON	P500	P750	P1000	P1500	SEM^3^	*P*_anova_	*P*_linear_	*P*_quadratic_
AhR1	0.94	0.93	0.91	1.31	1.15	0.187	0.379	0.066	0.806
ARNT	1.23	0.69	1.12	0.98	1.10	0.225	0.173	0.962	0.221
CYP1A1	1.04	0.93	0.86	1.05	1.39	0.312	0.587	0.251	0.172
CYP1A2	1.80	0.87	0.95	1.14	1.05	0.425	0.427	0.208	0.123
CYP1B1	1.68^A^	0.88^B^	1.18^AB^	0.96^B^	0.70^B^	0.232	0.007	0.001	0.364
GSTA2	0.55^B^	0.97^B^	1.17^AB^	1.89^A^	1.28^AB^	0.263	<0.001	<0.001	0.035
NQO1	0.88	0.98	1.23	1.21	1.13	0.148	0.109	0.036	0.120

1 PP Inclusion (CON = 0 mg/kg, P500 = 500 mg/kg, P750 = 750 mg/kg, P1000 = 1000 mg/kg, and P1500 = 1500 mg/kg of diet). Data represent treatment means from n = 7 replicates per treatment.

2 Means with different superscripts (a, b or A, B) within the same row differ significantly (*P* < 0.05 or 0.01).

3 Standard error of the means. AhR1, aryl hydrocarbon receptor 1; ARNT, aryl hydrocarbon receptor nuclear translocator; CYP1A1, cytochrome P450 1A1; CYP1A2, cytochrome P450 1A2; CYP1B1, cytochrome P450 1B1; GSTA2, glutathione S-transferase alpha 2; NQO1, NAD(P)H quinone dehydrogenase 1

#### Week 12

Dietary PP inclusion level decreased the expression of *CYP1B1* (*P* = 0.007) compared to CON, following a linear (*P*_lin_ = 0.001) pattern of decrease with increasing PP level. In addition, the expression of *GSTA2* differed (*P* < 0.001) between the experimental treatments, displaying a linear (*P*_lin_ < 0.001) and quadratic (*P*_quad_ = 0.035) pattern of increase with increasing PP level. Furthermore, a linear pattern of increase in *NQO1* (*P*_lin_ = 0.036) with increasing PP level was noted. The relative gene expression of *CYP1B1* was lowest for treatment P1500 and of *GSTA2* highest for P1000, always compared to control treatment.

### mRNA transcript Levels of Keap1/Nrf2/ARE pathway genes in Ovaries

#### Week 8

The expression levels of *Keap1/Nrf2/ARE* pathway (*Nrf2, Keap1, CAT, SOD1, GPX2, GPX7, GSR, PRDX1*, and *HMOX1*), and heat shock response (*HSP70* and *HSP90*) related genes at wk 8 of the experiment (28 wk of layers age) are presented in Table 5. The expression of *Nrf2* (*P* = 0.002), *Keap1* (*P* < 0.001), *SOD1* (*P* = 0.025), and *GSR* (*P* = 0.001) differed between the experimental treatments. Increasing PP inclusion level resulted in quadratic patterns of increase for *Nrf2* (*P*_quad_ = 0.009), *SOD1* (*P*_quad_ = 0.032), *GSR* (*P*_quad_ = 0.001) and decrease for *Keap1* (*P*_quad_ = 0.007). *SOD1, GPX7*, and *GSR* expression displayed linear patterns of increase (*P*_lin_ = 0.009; 0.050 and 0.002; respectively) and decrease for *Keap1* (*P*_lin_ < 0.001). Compared to CON, the relative expression levels of *SOD1* and *GSR* were highest for P1000. In the case of *Nrf2* and *Keap1,* P750 treatment had the highest expression and P2000 treatment had the lowest expression compared to CON, respectively.

**Table 5:** Relative gene expression of Nrf2 pathway and Heat Shock Response related genes in layer ovaries at 8th week (28 wk old layers) of the experiment.

Nrf2 pathway	Treatments^1^						Statistics^2^		
Genes (28 wk old layers)	CON	P500	P750	P1000	P1500	SEM^3^	*P*_anova_	*P*_linear_	*P*_quadratic_
Nrf2	1.04^B^	0.99^B^	1.57^A^	1.01^B^	0.95^B^	0.155	0.002	0.653	0.009
KEAP1	1.72^A^	1.04^B^	0.83^B^	0.83^B^	0.80^B^	0.199	<0.001	<0.001	0.007
CAT	0.86	1.14	1.68	1.45	0.95	0.461	0.366	0.631	0.065
SOD1	0.78^b^	1.12^ab^	1.28^ab^	1.35^a^	1.21^ab^	0.177	0.025	0.009	0.032
GPX2	0.94	1.22	0.97	1.81	1.02	0.560	0.770	0.755	0.555
GPX7	1.11	0.99	1.10	1.53	1.48	0.281	0.215	0.050	0.529
GSR	0.65^B^	1.08^AB^	1.41^A^	1.56^A^	1.17^AB^	0.196	0.001	0.002	0.001
PRDX1	0.85	1.21	1.25	1.14	1.27	0.210	0.269	0.107	0.282
HMOX1	0.96	1.11	1.22	1.02	1.12	0.189	0.689	0.594	0.415
**Heat shock response**									
HSP70	1.39	1.06	1.02	1.05	1.04	0.200	0.189	0.077	0.129
HSP90	1.17	1.00	1.15	1.20	1.53	0.250	0.321	0.107	0.185

1 PP Inclusion (CON = 0 mg/kg, P500 = 500 mg/kg, P750 = 750 mg/kg, P1000 = 1000 mg/kg, and P1500 = 1500 mg/kg of diet). Data represent treatment means from n = 7 replicates per treatment.

2 Means with different superscripts (a, b or A, B) within the same row differ significantly (*P* < 0.05 or 0.01).

3 Standard error of the means. CAT, catalase; Keap1, kelch-like ECH-associated protein 1; GPX2,7, glutathione peroxidase 2, 7; GSR, glutathione-disulfide reductase; HMOX1, heme oxygenase 1; HSP70, heat shock 70 kDa protein; HSP90, heat shock protein 90 alpha family class A member 1; Nrf2, nuclear factor; erythroid 2-like 2; SOD1, superoxide dismutase 1; PRDX1, peroxiredoxin-1.

#### Week 12

The results of the expression levels of *Keap1/Nrf2/ARE* pathway (*Nrf2, Keap1, CAT, SOD1, GPX2, GPX7, GSR, PRDX1*, and *HMOX1*), and heat shock response (*HSP70* and *HSP90*) related genes at week 12 of the experiment (32 wk of layers age) are shown in Table 6. Significant differences between the experimental treatments were shown for the expression of *SOD1* (*P* < 0.001), *GPX2* (*P* < 0.001), *GPX7* (*P* = 0.001), *GSR* (*P* < 0.001), *PRDX1* (*P* < 0.001), and *HMOX1* (*P* = 0.022). More specifically, *SOD1, GPX2, GSR, PRDX1*, and *HMOX1* expression in treatment P1000 were significantly higher compared firstly to CON and secondly to PP supplemented treatments*.* Furthermore, increasing PP inclusion level resulted in patterns of expression increase in a quadratic manner for *SOD1* (*P*_quad_ = 0.005), *GPX2* (*P*_quad_ = 0.006), *GPX7* (*P*_quad_ = 0.030), *PRDX1* (*P*_quad_ = 0.001), and *HMOX1* (*P*_quad_ < 0.001) and decrease in a quadratic manner for *Keap1* (*P*_quad_ = 0.041). The expression, of the majority of the *Keap1/Nrf2/ARE* pathway genes showed linear (*P*_lin_ ≤ 0.05) pattern of increase with increasing PP inclusion level with P1000 being higher than control treatment.

**Table 6:** Relative gene expression of Nrf2 pathway and Heat Shock Response related genes in layer ovaries at 12th week (32 wk old layers) of the experiment.

Nrf2 pathway	Treatments^1^						Statistics^2^		
Genes (32 wk old layers)	CON	P500	P750	P1000	P1500	SEM^3^	*P*_anova_	*P*_linear_	*P*_quadratic_
Nrf2	1.11	1.26	1.25	1.25	0.93	0.208	0.460	0.441	0.103
KEAP1	1.30	0.82	0.99	0.90	1.01	0.161	0.062	0.177	0.041
CAT	0.85	1.07	1.38	1.13	1.11	0.192	0.137	0.192	0.051
SOD1	0.48^B^	1.13^B^	1.25^B^	2.26^A^	1.16^B^	0.321	<0.001	0.002	0.005
GPX2	0.52^B^	0.95^B^	1.16^B^	2.46^A^	1.22^B^	0.288	<0.001	<0.001	0.006
GPX7	0.53^B^	1.01^B^	1.18^AB^	1.87^A^	1.24^AB^	0.281	0.001	0.001	0.030
GSR	0.81^B^	0.87^B^	0.87^B^	2.33^A^	1.37^B^	0.285	<0.001	<0.001	0.443
PRDX1	0.56^C^	1.04^B^	0.98^B^	2.12^A^	1.30^B^	0.194	<0.001	<0.001	0.001
HMOX1	0.57^b^	1.01^b^	1.14^b^	2.13^a^	0.96^b^	0.219	0.022	0.001	<0.001
**Heat shock response**									
HSP70	1.23	1.00	1.12	1.14	0.79	0.184	0.168	0.078	0.491
HSP90	1.17	0.85	1.05	1.30	0.93	0.187	0.151	0.930	0.918

1 PP Inclusion (CON = 0 mg/kg, P500 = 500 mg/kg, P750 = 750 mg/kg, P1000 = 1000 mg/kg, and P1500 = 1500 mg/kg of diet). Data represent treatment means from n = 7 replicates per treatment.

2 Means with different superscripts (a, b, c or A, B, C) within the same row differ significantly (*P* < 0.05 or 0.01).

3 Standard error of the means. CAT, catalase; GSR, glutathione-disulfide reductase; GPX2,7, glutathione peroxidase 2, 7; HMOX1, heme oxygenase 1; HSP70, heat shock 70 kDa protein; HSP90, heat shock protein 90 alpha family class A member 1; Keap1, kelch-like ECH-associated protein 1; Nrf2, nuclear factor; erythroid 2-like 2; PRDX1, peroxiredoxin-1; SOD1, superoxide dismutase 1.

## DISCUSSION

This study conducted under non-challenge experimental conditions, aimed to generate baseline physiological information on the AhR/Nrf2 genes response in the ovaries of laying hens fed diets with increasing phytogenic levels. An extensive palette of the AhR and Nrf2 critical genes relevant to detoxification and antioxidant capacity, including the expression of heat shock protein genes *HSP70* and *HSP90* was utilized.

In laying hens, productive performance and egg quality are strictly linked to ovarian performance. One of the most dominant factors that cause ovarian damage is oxidative stress which positively correlates with high productivity and the aging process (Finkel and Holbrook, 2000; Hao et al., 2021). Dysfunction and aging of the ovaries can lead to detrimental effects for poultry performance, reproductive activity, hatching rate, product quality and life span.

Growing evidence indicates that dietary phytogenics, in addition to their benefits for performance (Abdel-Wareth and Lohakare, 2020; Zhang et al., 2022), may also attenuate the oxidative stress as they regulate the gene expression of cytoprotective enzymes with detoxifying, antioxidant and anti-inflammatory functions (Köhle and Bock, 2006; Xun et al., 2021; Mountzouris et al., 2022). The latter involves the activation of two signaling pathways, namely aryl hydrocarbon receptor (AhR) and nuclear factor erythroid 2-related factor 2 (Nrf2). As a cytosolic xenobiotic sensor, AhR can be activated by a variety of exogenous and endogenous ligand such us dioxins, mycotoxins, phytochemicals and bacterial pathogens (Hernández-Ochoa et al., 2009; Liang et al., 2022). AhR ligands bind to AhRs multiprotein complex and get transferred into the nucleus. After the binding with AhR nuclear translocator (*ARNT*) the AhR-ARNT complex binds to xenobiotic-responsive element (**XRE**) and regulates the expression of xenobiotic-metabolizing enzymes (**XME**) or phase I enzymes. In particular, cytochrome P450 (**CYP**) enzymes (*CYP1A1, CYP1A2, CYP1B1*) are involved in Phase I metabolism, and their enzyme activity participates in detoxification of xenobiotics (Larigot et al., 2018; Mountzouris et al., 2022). In particular, transcription factors AhRs are responsible for the regulation of quinone oxidoreductase 1 (*NQO1*) and glutathione transferase A2 (*GSTA2*) enzymes, which link both AhR and Nrf2 pathways, displaying detoxifying and antioxidant properties (Köhle and Bock, 2006; Lee et al., 2018).

Moreover, Nrf2 is characterized as a crucial transcription factor, which regulates the cellular antioxidant response (Kovac et al., 2015). Under homeostatic conditions, Nrf2 binds to the Kelch-like ECH associating protein 1 (Keap1) keeping negatively regulated in the cytoplasm. Potential inducers such us phytogenics cause the disruption of Nrf2 and Keap1 cytoplasmic complex and allow Nrf2 to translocate into the nucleus (Sahin et al., 2013; Seymour et al., 2013). There it binds to antioxidant response element (**ARE**) and regulates the transcription of multiple cytoprotective genes known for their antioxidant (e.g., *CAT, SOD, GPX2, GPX7, GSR, PRDX1*), detoxifying (e.g., *GSTA2, NQO1*) and anti-inflammatory functions (e.g., *HMOX1*). (Sahin et al., 2013; Zerin et al., 2013; Stefanson and Bakovic, 2014; Ahmed et al., 2017).

In this work, the animal trial started when layers were in the beginning of laying and ended after 12 wk in the peak production phase (i.e., 20–32 wk of layers age). During the trial, performance responses were closely monitored on a weekly basis. When the differences in zootechnical performance responses became significant, layers were sampled and ovaries were collected for further analyses. In addition, layers were sampled at the end of the trial. Overall, phytogenic inclusion level improved significantly (*P* < 0.05) the laying rate and the egg mass for the growth periods 5 to 8 wk and 9 to 12 wk (25–28 and 29–32 wk of layers age, respectively). From the polynomial contrast analysis the phytogenic inclusion level of 1,000 mg/kg was identified as the most optimal with respect to the improvement of the laying rate and the egg mass.

This study demonstrated for the first time that dietary phytogenics can function as AhR pathway modulators in the ovaries of laying hens. In particular, PP inclusion reduced the expression of critical AhR pathway genes (*ARNT, CYP1A1, CYP1B1*) and increased the expression of *GSTA2*. Antos et al. (2015) have indicated that the liver is the main detoxification organ and the ovary only participates in this process. In our study, the observed decrease in the AhR related ovarian gene expression during the peak phase, suggested, that the detoxification requirements decreased with increasing PP inclusion level. It is also important to note that several studies have shown that the AhR plays a role in ovarian function by modulating estradiol production, follicle growth and ovulation process (Benedict et al., 2003; Valdez and Petroff, 2004; Barnett et al., 2007; Hernández-Ochoa et al., 2009). More specifically, activation of the AhR pathway accelerates reproductive senescence, possibly by disrupting ovarian, hypothalamic or suprachiasmatic nucleus function, or any combination of these (Valdez and Petroff, 2004).

Considering, the performance results and the above, it could be postulated that the downregulation of the AhR pathway by PP inclusion had a positive contribution to the reproductive process of laying hens. Additionally, reduced requirements for detoxification indicate better homeostatic conditions in the ovary. The latter could be an additional asset in protecting the developing embryo in the case of breeder hens.

In this work, in addition to the AhR pathway genes, PP inclusion modulated the expression of Nrf2 pathway genes. Phytogenic premix related cytoprotective potential was strongly supported via beneficial changes seen for the majority (9 out of 11) of the Nrf2-pathway genes assessed. The activation of the Nrf2 pathway upregulated the expression of many Phase II enzymes (e.g., *SOD1, GPX2, GPX7, GSR, PRDX1, HMOX1*), indicating a potential protective effect of PP against oxidative threat in the ovaries. Recent studies have demonstrated that aging can also trigger the oxidative stress resulting in lipid peroxidation and reduction of antioxidant capacity in the ovaries and liver tissues of laying hens, combined with down-regulation in the expression levels of some antioxidant genes (Liu et al., 2018a; Xing et al., 2020; Gu et al., 2021).

The animal capacity to mitigate oxidative stress is critical for the maintenance of egg production and hens’ health, especially in the prepeak period of laying, which can have long-lasting beneficial effects across the production period (Finkel and Holbrook, 2000; Eid et al., 2021; Durand et al., 2022). Furthermore, it has been recently shown that the PP inclusion used in this work resulted in a consistent expression increase of cytoprotective genes and improved intestinal total antioxidant capacity (**TAC**), as well as an enhanced liver, breast meat and thigh meat TAC in broilers (Mountzouris et al., 2019, 2020). Several studies (Mattson and Cheng, 2006; Mattson et al., 2007; Jodynis-liebert and Kujawska, 2020) have reported that many plant-derived bioactive compounds demonstrate dose-response properties and are considered to be hormetic compounds, that is, they induce biologically opposite effects at different doses.

It is interesting to note that in the ovaries, the down-regulated AhR and up-regulated Nrf2 pathway genes with increasing PP inclusion level, largely displayed quadratic patterns pointing to the 1,000 mg PP /kg diet as the most optimal. Therefore, under the physiologically nonchallenge experimental conditions used, PP inclusion level of 1,000 mg/kg diet was fit to improve layers performance and modulate the relevant gene expressions related to detoxification and antioxidant capacity.

Considering all the above together, PP inclusion has resulted in a consistent beneficial modulation of detoxification and cytoprotective responses that were concomitant with the improved performance findings.

In conclusion, this work has contributed further knowledge on phytogenic mechanisms of action at the ovaries level. It was shown that PP inclusion down-regulated the expression of genes relevant for detoxification and up-regulated the expression of critical genes for host protection against oxidation. These effects were concomitant with respective improvements in layer performance noted at the 1,000 mg PP/kg diet level. Future work could focus in investigating phytogenic cytoprotective effects in aged laying hens and under challenged conditions.
